# Protection induced by virus-like particles containing *Toxoplasma gondii* microneme protein 8 against highly virulent RH strain of *Toxoplasma gondii* infection

**DOI:** 10.1371/journal.pone.0175644

**Published:** 2017-04-13

**Authors:** Su-Hwa Lee, Ah-Ra Kim, Dong-Hun Lee, Ilaria Rubino, Hyo-Jick Choi, Fu-Shi Quan

**Affiliations:** 1 Department of Biomedical Science, Graduate School, Kyung Hee University, Seoul, Korea; 2 Department of Chemical and Materials Engineering, University of Alberta, Edmonton, AB, Canada; 3 Department of Medical Zoology, Kyung Hee University School of Medicine, Seoul, Korea; Food and Drug Administration, UNITED STATES

## Abstract

*Toxoplasma gondii* (*T*. *gondii*) microneme protein 8 (MIC8) represents a novel, functional distinct invasion factor. In this study, we generated virus-like particles (VLPs) targeting *Toxoplasma gondii* MIC8 for the first time, and investigated the protection against highly virulent RH strain of *T*. *gondii* in a mouse model. We found that VLP vaccination induced *Toxoplasma gondii-*specific IgG and IgG1 antibody responses in the sera. Upon challenge infection with RH strain of *T*. *gondii* tachyzoites, vaccinated mice showed a significant increase of both IgG antibodies in sera and IgA antibodies in feces compared to those before challenge, and a rapid expansion of both germinal center B cell (B220^+^, GL7^+^) and T cell (CD4^+^, CD8^+^) populations. Importantly, intranasally immunized mice showed higher neutralizing antibodies and displayed no proinflammatory cytokine IFN-γ in the spleen. Mice were completely protected from a lethal challenge infection with the highly virulent *T*. *gondii* (RH) showing no body weight loss (100% survival). Our study shows the effective protection against *T*. *gondii* infection provided by VLPs containing microneme protein 8 of *T*. *gondii*, thus indicating a potential *T*. *gondii* vaccine candidate.

## Introduction

Toxoplasmosis represents a parasite disease caused by *T*. *gondii*, which is estimated to infect half of the world’s population [1]. In case of people with weakened immune systems, due to conditions such as AIDS and cancer or immunosuppressant medication, toxoplasmosis is deadly. Additionally, infection of livestock represents a great economic burden and a source of transmission to humans, causing foodborne disease outbreaks [2–4]. Currently, no effective treatment of toxoplasmosis is available, raising the need to develop a vaccine for protection against *T*. *gondii* [5]. However, no human or veterinary vaccine against *T*. *gondii* has been licensed to date.

For this purpose, recombinant protein vaccines have been extensively investigated as a potential vaccine candidate using surface antigens [6,7], rhoptry antigens [8,9], dense granule proteins [7,10] and microneme proteins [11–13]. However, these vaccine candidates exhibited limited efficacy, thus failing to offer complete protection against infection. As a result, great efforts have been aimed to exploiting new approaches to develop effective *T*. *gondii* vaccines. Among the reported recombinant proteins, particular attention has been focused on the microneme proteins as potential vaccine antigens because of their critical involvement in host cell invasion [14–16]. Previous studies have reported partial protection with an individual *T*. *gondii* microneme protein 1 (TgMIC1), TgMIC4, and TgMIC6, or combinations thereof [16]. In addition, *T*. *gondii* MIC8, a protein expressed in tachyzoites which functions as an escorter for soluble adhensins to the cells, has been recognized as a potential vaccine candidate against acute and chronic *T*. *gondii* infection [13,17].

We have previously demonstrated the protective efficacy of virus-like particles (VLPs) containing inner membrane complex (IMC) by measuring 100% survival rate of vaccinated mice upon challenge infection [5]. However, it should be noted that ME49, a moderately virulent strain of *T*. *gondii*, was employed for challenge. Thus, it is critical to evaluate the effectiveness of different types of VLP vaccines against highly the virulent RH strain of *T*. *gondii*. Moreover, MIC8 of *T*. *gondii* is known to play a critical role in host cell invasion by the parasite [18]. Therefore, it is hypothesized that *T*. *gondii* VLPs targeting MIC8 would elicit *T*. *gondii*-specific humoral and cellular immune responses, resulting in the induction of protective immunity against toxoplasmosis.

In this study, we report the development of VLPs to target *T*. *gondii* MIC8 for the first time, and the evaluation of their protective efficacy against infection of highly virulent *T*. *gondii* (RH) in a mouse model. We found that VLP vaccination showed *T*. *gondii*-specific humoral and cellular immune responses, no proinflammatory cytokine IFN-γ in the spleen, and mice were completely protected from lethal challenge.

## Materials and methods

### Ethics statement

All animal experiments and husbandry involved in these studies were conducted under the guidelines of the Kyung Hee University IACUC. Kyung Hee IACUC operates under the National Veterinary Research and Quarantine Service (NVRQS) and regulations of the World Organization for Animal Health (WOAH). All animal procedures performed in this study (permit number: KHUASP (SE)– 16–012) were reviewed, approved, and supervised by an animal research ethics committee in Kyung University. The research staff was trained in animal care and handling, and received the certificate of completion for Animal Welfare & Ethics Course (K-2015-18060371) from the CITI. All surgery was performed under Isoflurane anesthesia (BSL 2), and all efforts were made to minimize suffering.

### Animals, parasites, cells and antibodies

Six-week-old female BLAB/c mice were obtained from NARA Biotech (Seoul, Korea). *Toxoplasma gondii* RH and ME49 strains were maintained according to the methods described previously [19–21]. *Spodoptera frugiperda* Sf9 cells used for production of recommended baculovirus (rBV) and virus-like particles were maintained in serum-free SF900 II medium (Invitrogen, Carlsbad, USA) in spinner flasks at 27°C and 130–140 rpm. Horseradish peroxidase (HRP)-conjugated goat anti-mouse immunoglobulin A (IgA), IgG, IgG1, and IgG2a were purchased from Southern Biotech (Birmingham, AL, USA).

### Preparation of *Toxoplasma gondii* antigen

Mice were infected with *Toxoplasma gondii* (RH) and tachyzoites were harvested from the mice peritoneal cavity 4 days after infection by injection of 2 mL of 0.1 M phosphate-buffered saline (PBS, pH 7.2) [22]. The exudate was separated from cellular debris by low speed centrifugation (100 g, 5 min) at 4°C. The parasites in the supernatant were precipitated by centrifugation at 600 g for 10 min, followed by washes in PBS and sonication on ice. After measuring the protein concentration using QuantiPro BCA Assay Kit (Sigma-Aldrich, St Louis, USA), *T*. *gondii* antigen was stored at –20°C until used.

### Construction of recombinant baculovirus (rBV) expressing *Toxoplasma gondii* microneme protein 8 (MIC8) and influenza matrix protein 1 (M1)

To clone the *T*. *gondii* MIC8 gene into the baculovirus expression vector (pFastBac), the total RNA was extracted from the *T*. *gondii* RH strain using RNeasy Mini Kit (Qiagen, Valencia, USA). The total RNA was reversely transcribed to cDNA using Prime Script 1^st^ strand cDNA synthesis kit according to the manufacturer’s instructions (Takara, Otsu, Japan). The cDNA was used as a template to amplify the complete coding sequence *T*. *gondii* MIC8 by polymerase chain reaction (PCR). The primers were designed from the nucleotide sequence of MIC8 in GenBank (accession number: AF353165): forward (AAAGAATTCACCATGAAGGCCAATCGAATATG) and reverse (TTACTCCAGTTAGGACCAGATACCGCCCGA) with *EcoR*I and *Xho*I restriction enzyme sites (underlined). The PCR product was inserted into the pFastBac plasmid (Invitrogen, Carlsbad, USA). For M1 gene cloning, whole procedure was followed as described [23]. The constructs containing M1 (accession number: EF467824, 1.027bp) and MIC8 (accession number: AF353165, 2.055bp) genes in the pFastBac vector were confirmed by DNA sequencing. The recombinant plasmids were transformed into a DH10-Bac, extracted using FavorPrep gel purification Kit (Favorgen, Cheshire, UK), and stored at –20°C until used.

### Production of recombinant baculovirus (rBV) and virus-like particles (VLPs)

Cellfectin II reagent (Invitrogen) was used for transfection of the DNAs with the genes described above into Sf9 cells. Subsequently, the pFastBac construct containing either *T*. *gondii* MIC8 or influenza M1 was transformed using blue/white screening, and rBVs were produced with a Bac-to-Bac expression system (Invitrogen). To produce VLPs containing MIC8 and M1, Sf9 cells were co-infected with rBVs expressing MIC8 and M1. Cell culture supernatants were collected on day 3 post-infection, cleared by centrifugation at 6,000 rpm for 30 min at 4°C to remove cells. VLPs in the supernatants were pelleted by high-speed centrifugation (45,000 g for 30 min). The sedimented particles were resuspended in 0.1 M PBS at 4°C overnight and further purified through a 20-30-60% discontinuous sucrose gradient at 45,000 g for 1 h at 4°C. The VLP bands were collected and pelleted by high-speed centrifugation (45,000 g for 30 min). 500 μL 0.1 M PBS were added to the VLPs to resuspend them by incubation overnight at 4°C. Band protein concentration was measured using QuantiPro BCA Assay Kit (Sigma-Aldrich, St Louis, USA). VLPs were stored at 4°C until used.

### Characterization of VLPs

To characterize VLPs, Western blots and transmission electron microscopy (TEM) were performed. The presence of MIC8 protein was detected by Western blot analysis in mouse serum, which was collected at week 4 from BALB/c mice infected with *T*. *gondii* ME49 strain. The amount of M1 protein was determined with monoclonal mouse anti-M1 antibody. Morphology of negatively stained VLPs was characterized by TEM at 200kV (JEOL 2100, JEOL USA, Inc.; Peabody, MA, USA) [24].

### VLPs immunization and challenge infection

Three mice groups (female, BALB/c mice; 20 mice in each group) were used in this work: intranasal immunization (IN), intramuscular immunization (IM) and non-immunized control. In our previous study, we have found that HA-negative M1 VLP-immunized mice did not show PR8-specific antibodies [25]. We assume that VLPs not expressing MIC8 protein may not induce any immune responses. Mice were immunized intranasally or intramuscularly with 75 μg of total MIC8 VLP protein per mouse at weeks 0 and 4. Four weeks after the last immunization, mice were challenged by oral administration with 1 × 10^5^ tachyzoites of the RH strain. Ten mice from each group were anesthetized (isoflurane), sacrificed on days 5 (6 mice in each group) or 16 (6 mice in each group) post-challenge, and blood and spleen samples were collected. The remaining mice (8 mice in each group) were observed daily to monitor changes in body weight and survival rates for 16 days post-challenge. Mice lost 20% in body weight were considered dead and humanly euthanized, which usually needs 12 days. Some mice died before meeting criteria. The euthanasia was performed by cervical dislocation under anesthetic condition using isoflurane by inhalation. Twenty-eight days (8 weeks) were needed for VLP immunization, 33 days (8wks+5d) for scarification, and 72 days (10wks+2d) were needed for termination of experiment.

### Antibody responses in sera and feces

The retro-orbital plexus puncture method was used to collect blood samples from mice at weeks 1, 2 and 4 after prime and boost, and on day 5 post-challenge. Sera were collected from the blood samples and stored at –20°C for evaluation of antibodies. Feces samples of mice were collected at week 4 after boost and on days 1–7 post-challenge. PBS was added to the feces samples for incubation at 37°C for 1 h. Subsequently, the feces samples were centrifuged at 2,000 rpm for 10 min, and the supernatants were harvested and stored at –20°C. IgG, IgG1, IgG2a and IgA antibodies were quantified by enzyme-linked immunosorbent assay (ELISA). To measure *T*. *gondii*-specific antibodies, 96 well flat-immunoplates were coated overnight at 4°C with 100 μL of *T*. *gondii* antigen at a final concentration of 0.2 μg/mL in 0.05 M pH 9.6 carbonate bicarbonate buffer per well. 100 μL of serum samples (diluted 1:100 in PBST) per well were incubated in the plates for 1.5 h at 37°C prior to the measurement of IgA in feces. HRP-conjugated goat anti-mouse IgA, IgG, IgG1 and IgG2a in PBST (100 μL/well) were used to determine *T*. *gondii*-specific antibody responses.

### Antibody neutralization

Mouse sera collected at week 4 after boost were complement inactivated at 56°C for 30 min and 50 μL of sera from IM or IN immunization were incubated with 100 tachyzoites of *T*. *gondii* (RH) at 37°C for 1 h. The *T*. *gondii*-serum mixture for IM and IN and *T gondii*-PBS control were used to intraperitoneally infect naive mice (10 mice in each group), including naïve mice infection control. At 7 days after infection, tachyzoites of *T*. *gondii* were collected from the abdominal cavity of the mice and counted by hemocytometer chamber under microscopy [26,27].

### T and B cell responses by flow cytometry

The percentages of T cell (CD4^+^, CD8^+^) and B cell within germinal center from splenocytes of mice on day 5 and 16 after challenge infection were analyzed by flow cytometry. Splenocytes (1 × 10^6^ cell/mL) in staining buffer (2% bovine serum albumin and 0.1% sodium azide in 0.1 M PBS) were incubated for 15 min at 4°C with Fc Block (clone 2.4G2; BD Biosciences, CA, USA). For surface staining, cells were incubated in the surface antibodies (CD3e-PE-Cy5, CD4-FITC, CD8a-PE, B220-FITC, GL7-PE; BD Biosciences) for 30 min at 4°C. Splenocytes were washed with staining buffer and fixed with 4% paraformaldehyde for 30 min at 4°C before acquisition using a FACSCalibur flow cytometer (BD Biosciences). Data were analyzed using Cell Quest Pro software (BD Biosciences).

### Inflammatory cytokine analysis

Spleen samples were collected at day 5 post-challenge to determine cytokine responses to challenge infection. Splenocytes from suspension were washed and 1 × 10^6^ cells/well were prepared in RPMI-1640 culture media supplemented with 10% FBS to culture splenocytes *in vitro*. Then, cytokine IFN-γ and IL-6 levels were determined by BD OptEIA Set (BD Biosciences) using the culture supernatants harvested after 5 days. Cytokine concentration was quantified by ELISA (λ = 490 nm, n = 3) with reference to standard curves constructed with BD IFN-γ and IL-6 standard stock solutions.

### Statistics

The data were analyzed statistically using the ANOVA with multiple comparison test of PC-SAS 9.3 (SAS Institute; Cary, NC, USA). A *P* value < 0.05 and < 0.01 was considered to be significant.

## Results

### Generation of constructs

**Fig 1:**
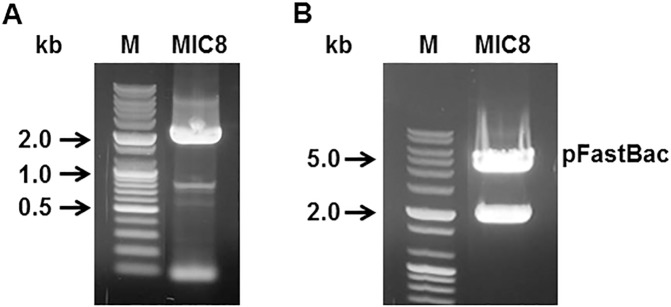
PCR identification of MIC8 and M1 genes and recombinant plasmids pFastBac-MIC8 and pFastBac-M1 digested. A Prime Script 1^st^ Strain cDNA Synthesis Kit and total RNA extracted from *T*. *gondii* RH strain were used to synthesize cDNA, which was then amplified by PCR to obtain *T*. *gondii* MIC8 gene (A). MIC8 plasmid (B) was obtained by cloning *T*. *gondii* MIC8 gene into pFastBac vector with *EcoR*I / *Xho*I enzymes. Marker: DNA marker; Size of MIC8: 2055bp.

*Toxoplasma gondii* microneme protein 8 (MIC8) was amplified by PCR with primers containing restriction enzyme sites (Fig 1A). The MIC8 gene was cloned into pFastBac vector. Using restriction enzymes *EcoR*I / *Xho*I, the insertion of *T*. *gondii* MIC8 in pFastBac expressing vectors was confirmed (Fig 1B). Influenza M1 was PCR amplified and cloned into pFastBac. The nucleotide sequence of the *T*. *gondii* MIC8 and influenza M1 genes were identical to previous reports (accession numbers AF353165.1 for *T*. *gondii* MIC8 and EF467824 for M1) by DNA sequencing (Eurofins MWG Operon).

### Production of virus-like particles (VLPs)

**Fig 2:**
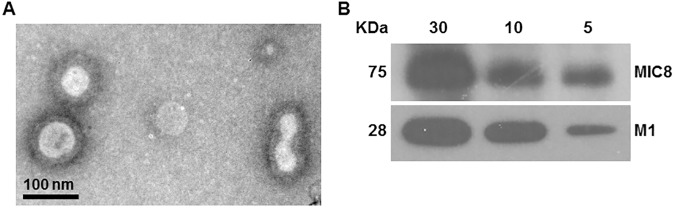
VLPs characterization. TEM image (A) and Western blot analysis (B). VLPs (5, 10, 30 μg) were loaded for SDS-PAGE. Polyclonal mouse anti-*T*. *gondii* antibody and anti-M1 monoclonal antibody were used to probe MIC8 protein and M1 protein, respectively.

The protocol described in the Methods section was used to produce VLPs containing *T*. *gondii* MIC8 and influenza M1. As shown in the TEM micrograph of Fig 2A, *T*. *gondii* MIC8 VLPs had a spherical morphology with approximate size of 40–120 nm, and exhibited antigen spikes on their surfaces. The results of Western blot analysis with anti *T*. *gondii* polyclonal antibody and M1 monoclonal antibody are displayed in Fig 2B, which confirms the incorporation of both *T*. *gondii* MIC8 and influenza M1 into VLPs.

### *T*. *gondii-*specific IgG, IgG1 and IgG2a antibody responses after immunization

**Fig 3:**
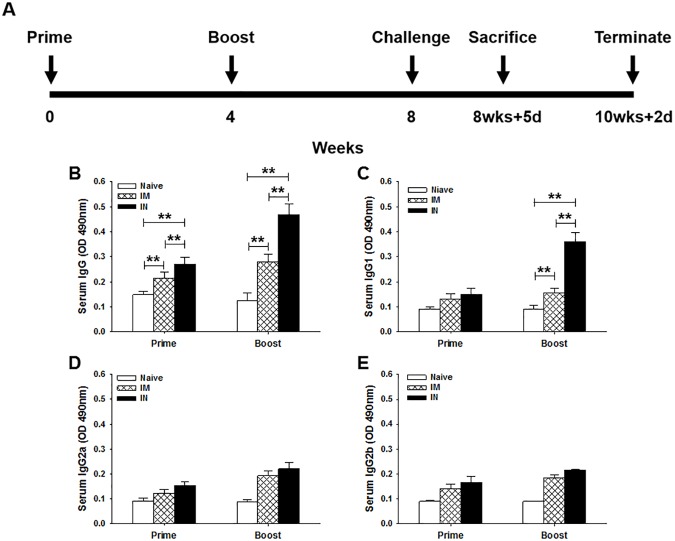
Experimental schedule and *T*. *gondii-*specific antibody responses upon immunization. Mice were immunized twice (boost VLP vaccination administered 4 weeks after the prime dose) and challenge infection was performed 4 weeks after boost vaccination. Mice were sacrificed at day 5 post-challenge (A). High levels of *T*. *gondii*-specific IgG, IgG1 and IgG2a antibody responses in the sera were determined after boost with ELISA assay (B, C, D, **P < 0.01). Lower levels of IgG2a and IgG2b were determined in IN mice compared to IgG1 (D, E). IM mice showed low levels of isotypes IgG1, IgG2a and IgG2b responses (C, D, E).

**Fig 4:**
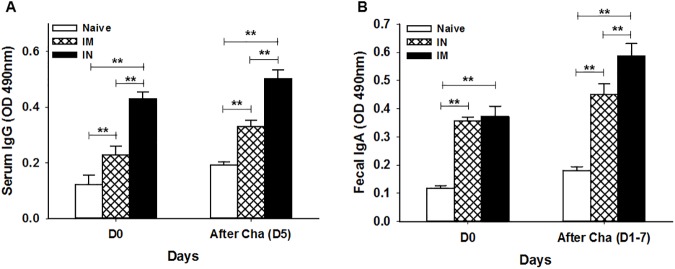
*T*. *gondii-*specific antibody response profiles in serum and feces following challenge infection. Immunized mice were challenged orally with *T*. *gondii* RH strain 4 weeks after boost vaccination, and *T*. *gondii*-specific IgG antibody responses in the sera were detected at day 5 after challenge infection (A; **P < 0.01). IgA antibody responses from feces were also determined after challenge infection (B; **P < 0.01).

To evaluate the level of *T*. *gondii*-specific antibody induced by MIC8 VLP vaccine, we measured the total IgG, IgG1, IgG2a and IgG2b antibody responses upon prime and boost in mice after IN or IM immunizations (Fig 3A). IN mice group showed higher levels of *T*. *gondii*-specific IgG antibody response compared to IM mice group (Fig 3B for IgG). IN mice group showed higher levels of *T*. *gondii*-specific IgG1 antibody response compared to IgG2a and IgG2b antibody responses (Fig 3C for IgG1, Fig 3D for IgG2a, Fig 3E for IgG2b), indicating that IN administration induced Th2-dominant responses. IM mice showed low levels of IgG isotypes IgG1 (Fig 3C), IgG2a (Fig 3D) and IgG2b (Fig 3E) responses, indicating that intramuscular route could not induce effective antibody responses. The antibody response profiles in serum and feces following challenge infection were determined by orally infecting mice groups with *T*. *gondii* RH strain at week 4 after boost. Higher levels of *T*. *gondii*-specific IgG or IgA antibodies were detected in serum at day 5 upon challenge or feces during days 1 to 7 upon challenge (Fig 4A and 4B; **P < 0.01) in IN mice groups. These results indicate that IgG and IgA antibodies were rapidly boosted by challenge infection with *T*. *gondii*, indicating IN administration induced higher levels of systemic and mucosal antibody responses upon challenge infections.

### Neutralizing antibody response

**Fig 5:**
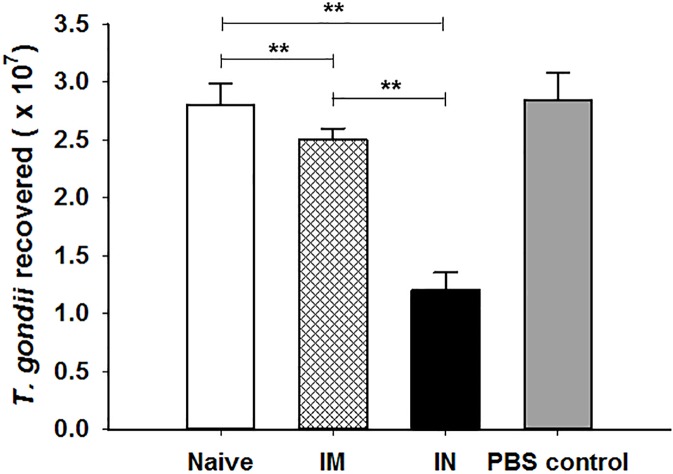
Serum neutralizing activities. Complement-inactivated mouse sera at week 4 after boost were used to react with *T*. *gondii* (RH) *in vitro*, and the mixture of sera (50 μL) and *T*. *gondii* (100 tachyzoites) was used to infect mice, including PBS and naïve mouse sera controls. At day 7 after infection, tachyzoites were recovered from mouse abdominal cavity and the replication inhibition of *T*. *gondii* was determined (**P < 0.01).

Neutralizing antibody is an important functional component of immune responses induced by vaccination. The mixtures of immune sera and tachyzoites were infected into mice, and 7 days later, tachyzoites from abdominal cavities of mice were calculated. As shown in Fig 5, significant reduction of tachyzoites was found in IN mice compared to IM, naïve and PBS control groups (**P < 0.01). These results indicate that neutralizing antibodies from IN mice neutralize the biological effects of the *T*. *gondii* tachyzoite.

### T and B cell responses upon challenge infection

**Fig 6:**
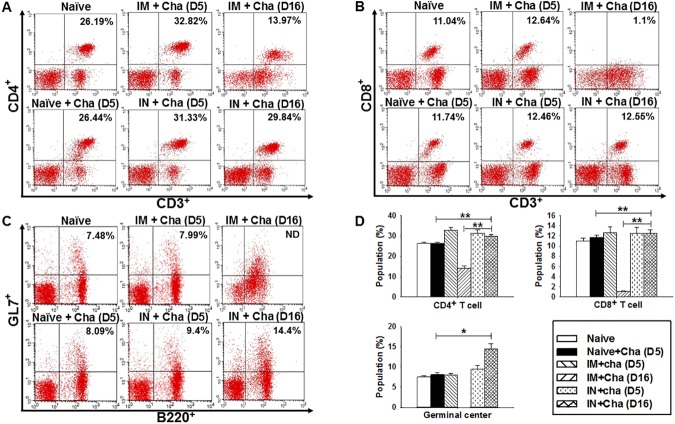
CD4^+^, CD8^+^ T and B cell responses after challenge infection. The populations of (A) CD4^+^ T cells, (B) CD8^+^ T cells and (C) germinal center B cells were analyzed at days 5 and 16 upon challenge using flow cytometry, and the results are summarized (D). Numbers indicate percentage of cell populations in each quadrant. Higher populations of CD4^+^, CD8^+^ and germinal center B cells were detected in IN+Cha mice (A, B, C, D, *P < 0.05, **P < 0.01).

To determine T cell (CD4^+^ and CD8^+^) and B cell (germinal center: GC) responses in immunized mice upon challenge, FACS analysis was performed using mouse spleen cells. As seen in Fig 6, at day 5 post-challenge, higher populations of CD4^+^, CD8^+^ T cells and germinal center B cells were found in IN (IN+Cha: intranasally immunized mice challenged with *T*. *gondii* RH) and IM (IM+Cha: intramuscularly immunized mice challenged with *T*. *gondii* RH) mice groups compared to non-infected naïve and non-immunized control groups (Naive+Cha: naïve mice challenged with *T*. *gondii* RH). Most importantly, at day 16 post-challenge, significantly higher populations of germinal center B cells (14.4%), CD4^+^ T cells (29.84%) and CD8^+^ T cells (12.55%) were observed in IN+Cha mice group compared to that in IM+Cha group (Fig 6A, 6B, 6C and 6D, *P < 0.05, **P < 0.01). Since all Naïve+Cha and 40% of IM+Cha mice groups were dead at about day 12 upon challenge, only IN+Cha mice group showed clear populations of germinal center B, CD4^+^ and CD8^+^ T cells. These results indicate that IN administration of VLP vaccine showed a significant increase of germinal center B cells, CD4^+^ and CD8^+^ T cells, which can contribute to the complete protection upon challenge infection.

### Proinflammatory cytokine response in splenocytes

**Fig 7:**
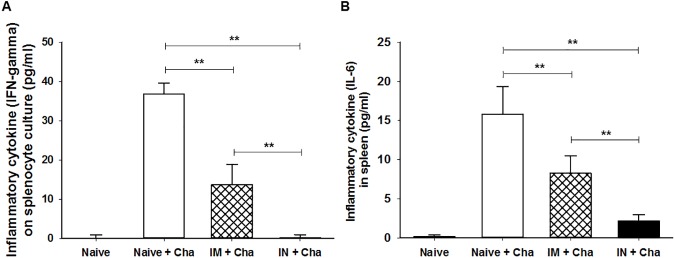
Proinflammatory cytokine response in the spleen. To determine the level of inflammatory cytokine IFN-γ and IL-6, mouse spleen cells were cultured *in vitro* for 5 days. Higher levels of IFN-γ and IL-6 were detected in Naïve+cha and IM+cha compared to the non-infected naïve mice (*P < 0.05). No IFN-γ were detected in IN+Cha mice (A, **P < 0.01). Significantly lower level of IL-6 were detected in IN+Cha mice compared to IM+Cha and Naïve+Cha mice (B, **P < 0.01).

Splenocytes were cultured *in vitro* in RPMI 1640 media for 5 days, to determine the amounts of inflammatory cytokine IFN-γ and IL-6 produced. As shown in Fig 7, higher levels of IFN-γ and IL-6 were detected in Naïve+Cha (IFN-γ: 36.8 pg/mL, IL-6: 15.79 pg/mL) and IM+Cha (IFN-γ: 13.6 pg/mL, IL-6: 8.25 pg/mL) compared to IN+Cha (IFN-γ: 0.17 pg/mL, IL-6: 2.14 pg/mL) and naïve control (IFN-γ: 0 pg/mL, IL-6: 0.155 pg/mL) (**P < 0.01). This suggests that IN immunized mice exhibited a completely reduced inflammatory reaction following challenge infection. The results exactly correlate with higher levels of antibody responses, germinal center B cells and CD4^+^ T cell responses in IN mice groups than in IM mice groups. These immune parameters are presumed to contribute to the complete protection observed in the intranasally immunized mice group.

### Complete protection induced by IN-administered VLPs upon challenge infection with *T*. *gondii* (RH)

**Fig 8:**
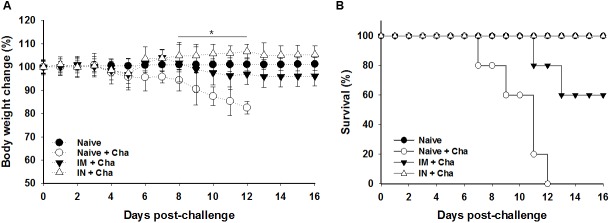
Survival rate and body weight changes upon challenge infection. Mice that were immunized intranasally or intramusculary with MIC8 VLPs were challenged with *T*. *gondii* RH strain. Body weight loss (A) and survival rates of mice (B) were monitored for 16 days. Significant difference was found in body weight loss at 8–12 days post-challenge between IN+cha and Naïve+cha (* *P* < 0.05).

To determine the protective efficacy of VLP vaccine, the immunized and control groups were challenged with lethal *T*. *gondii* RH strain (1 × 10^5^ tachyzoites) 4 weeks after boost. Body weight changes and survival rates were monitored for 16 days. As shown in Fig 8A and 8B, IN mice group showed 100% protection without any body weight loss, whereas IM mice group exhibited partial protection, resulting in 60% survival and 4% body weight loss. Mice in control group showed body weight loss of 17.5%, followed by death before day 12 upon challenge (Fig 8A and 8B). These results indicate that IN immunization of mice with MIC8 VLPs provided complete protection against lethal challenge infection with the highly virulent *T*. *gondii* (RH).

## Discussion

In the present study, we generated *T*. *gondii* VLPs to target MIC8, and the efficacy of VLP vaccine was tested by challenging immunized mice with highly virulent *T*. *gondii* RH. Vaccination elicited significantly higher levels of T and B cell responses. Importantly, IN vaccination with VLPs conferred complete protection (100% survival) against lethal challenge infection with *T*. *gondii* (RH).

VLPs have shown remarkable effectiveness as vaccine candidates in virus vaccine research [28]. A crucial benefit of VLPs is their safety due to a structure that is designed to overall resemble the original virus, but with the exclusion of the virus genomic material [28,29]. Here, we used influenza matrix M1 as a core protein to generate *T*. *gondii* VLPs where repeated structure of foreign microneme protein 8 (MIC8) from *T*. *gondii* was supposed to be formed on the surface of VLPs. As shown in Fig 2A, M1 core protein conferred a spherical particle shape to the VLP morphology. In addition, MIC8 in VLPs could play an important role in inducing humoral or cellular immunity, and complete protection (Fig 8A and 8B).

In the production of new vaccines, mucosal immunization is regarded as a promising strategy because of its capability to induce both mucosal and systemic immune responses [30]. IN vaccination can induce IgG and IgA antibody responses in respiratory, intestinal and genital mucosa [31,32]. As such, in this work, IN immunization with VLPs was proven to elicit IgA antibody responses in intestinal mucosa (feces) as well as IgG antibody responses in sera, indicating the induction of both mucosal and systemic responses. Interestingly, IN immunization induces higher neutralizing antibody responses compared to IM, indicating that immune sera from IN immunization are protective. Importantly, upon challenge, IgA antibody in feces of IN mice group showed a significant increase compared to IgG antibody in sera (Fig 4A and 4B), implying an important role of mucosal immunity. Considering the defense mechanism of IgA in epithelial cell line [33], we believe that IN immunization successfully inhibited the replication of *T*. *gondii*, resulting in 100% survival without any body weight loss. We also observed higher populations of CD4^+^ and CD8^+^ T and germinal center B cells in IN mice group compared to IM mice group at day 16 post-challenge (CD4^+^ T: 29.8% vs 13.97%; CD8^+^ T: 12.55% vs 1.1%; GC: 14.4% vs ND). This is consistent with the measurement of higher levels of IgG, IgG1, IgG2a and IgA antibody responses in IN mice group than in IM mice group, further supporting the complete protection observed in IN mice group.

Germinal centers (GC) are the main sites where antigen‐activated B‐cell clones expand [34]. B cells in germinal center differentiate into either memory B cells or antibody-secreting plasma cells [35]. Germinal center B cells have been reported to respond rapidly on re-infection with *Plasmodium chabaudi* malaria, and contribute to the development of memory B cells and their differentiation into plasma cells [36]. Thus, we assessed the number of germinal center B cells in IN mice group, and found a significant expansion of GC population at days 5 and 16 upon challenge compared to non-immunized controls (D5: 9.4% vs 8.1%; D16: 14.4% vs 8.1%). These observations explain the complete protection induced in IN mice group. On the other hand, partial protection (60% survival) of IM mice group can be accounted for by no noticeable change in population of GC compared to non-immunized mice group (D5: 8% vs 8.1%).

Infection with *T*. *gondii* RH tachyzoites led to remarkably high levels of IFN-γ production in the spleen. Notably, *T*. *gondii*-induced overproduction of proinflammatory cytokine IFN- γ can cause pathologic effects and death [37]. We observed significantly higher levels of IFN-γ in the spleen of naïve control and IM mice upon challenge, in contrast to no IFN-γ in IN mice group (Fig 7). These results indicate that IN immunization can avoid proinflammatory cytokine production upon challenge, leading to the protection of mice from tissue damage and death [38].

In summary, our results demonstrate that VLP vaccine containing *T*. *gondii* MIC8 can induce humoral and cellular immune responses, which can confer complete protection against highly virulent *T*. *gondii* RH tachyzoite infection. In addition, mucosal IgA antibody and germinal center B cell responses induced by IN immunization were increased upon challenge infection, inhibiting tachyzoite replication and spleen inflammatory cytokine production. These results provide an effective approach for developing vaccines based on VLPs for protection against the highly virulent RH strain of *T*. *gondii*.

## Supporting information

S1 Fig Survival monitoring. To determine the dose of VLP vaccine for mouse immunization, mice were intranasally immunized with 60 μg of total MIC8 VLP protein per mouse at weeks 0 and 4. Four weeks after the last immunization, mice were challenged by oral administration with 1 × 10^5^ tachyzoites of the RH strain. The mice (10 mice in each group) were observed daily to monitor changes in body weight (A) and survival rates (B) for 17 days post-challenge. As a result, it was confirmed that mice immunized with 60 μg of total MIC8 VLP failed to induce complete protection against *Toxoplasma gondii* infection, showing 20% of survival.
(TIF)

S1 Table ARRIVE guideline checklist. (DOCX)
