# Supplementary figures and images for: Protection induced by virus-like particles containing Toxoplasma gondii microneme protein 8 against highly virulent RH strain of Toxoplasma gondii infection
Source: PLoS One. 2017 Apr 13;12(4):e0175644. doi: 10.1371/journal.pone.0175644 (PMC5391012; doi:10.1371/journal.pone.0175644)

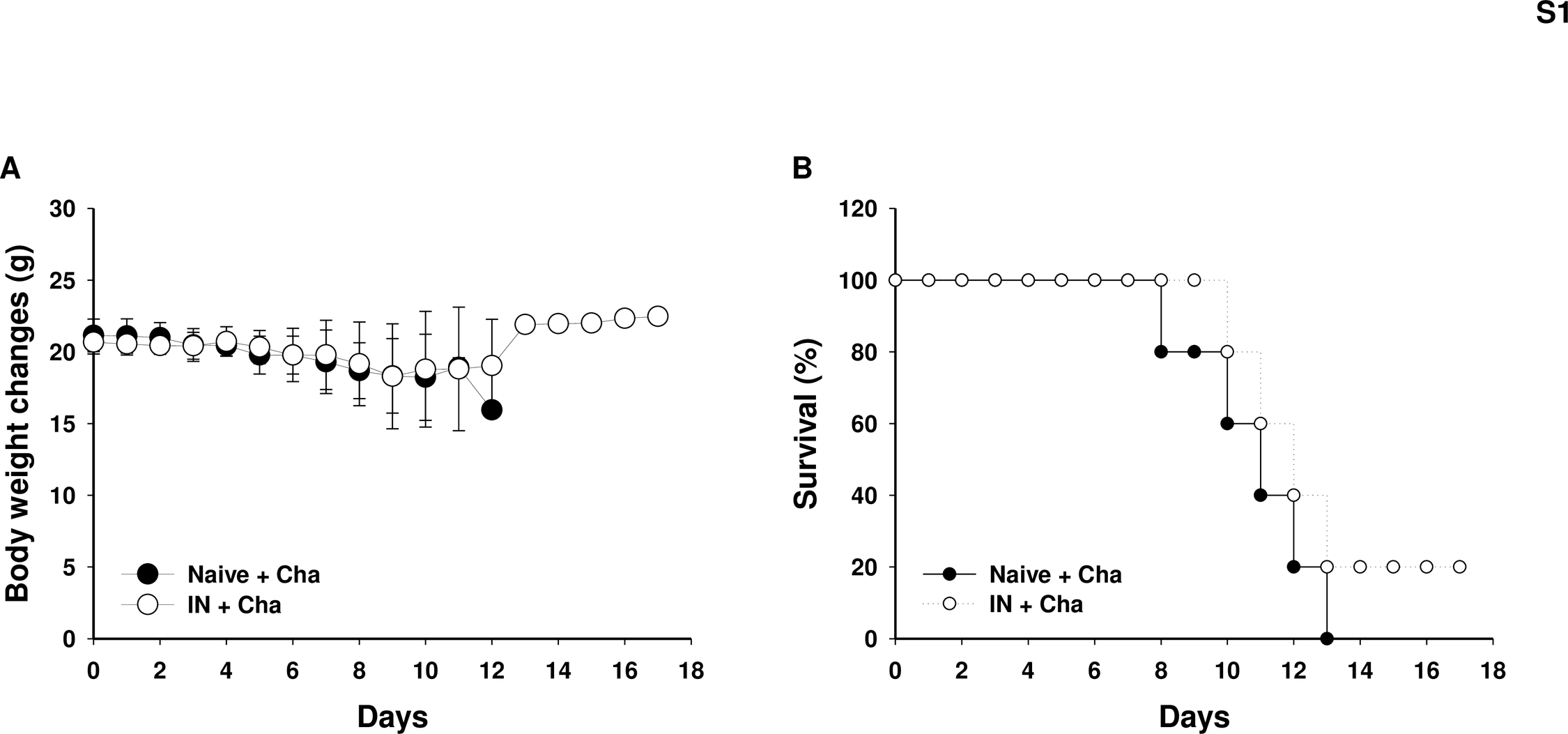

Supplement: S1 Fig — To determine the dose of VLP vaccine for mouse immunization, mice were intranasally immunized with 60 μg of total MIC8 VLP protein per mouse at weeks 0 and 4. Four weeks after the last immunization, mice were challenged by oral administration with 1 × 10^5^ tachyzoites of the RH strain. The mice (10 mice in each group) were observed daily to monitor changes in body weight (A) and survival rates (B) for 17 days post-challenge. As a result, it was confirmed that mice immunized with 60 μg of total MIC8 VLP failed to induce complete protection against Toxoplasma gondii infection, showing 20% of survival. (TIF) [file pone.0175644.s001.tif]
