# Supplementary material for: Protection induced by virus-like particles containing Toxoplasma gondii microneme protein 8 against highly virulent RH strain of Toxoplasma gondii infection
Source: PLoS One. 2017 Apr 13;12(4):e0175644. doi: 10.1371/journal.pone.0175644 (PMC5391012; doi:10.1371/journal.pone.0175644)
Supplement: S1 Table — (DOCX) [file pone.0175644.s002.docx]

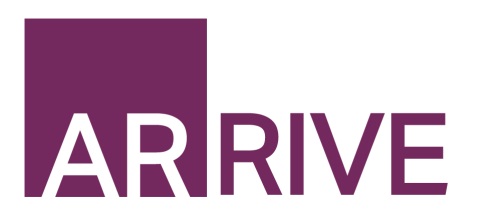


The ARRIVE Guidelines Checklist

Animal Research: Reporting In Vivo Experiments

Su-Hwa Lee^1^, Ah-Ra Kim^1^, Dong-Hun Lee^1^, Ilaria Rubino^2^, Hyo-Jick Choi^2^, Fu-Shi Quan^3*^

*^1^The 1Department of Biomedical Science, Graduate School, Kyung Hee University, Seoul, 130-701, Korea, ^2^Department of Chemical and Materials Engineering, University of Alberta, Edmonton, AB T6G 1H9, Canada, ^3^Department of Medical Zoology, Kyung Hee University School of Medicine, Seoul, 130-701, Korea*

|  | | ITEM | RECOMMENDATION | Section/ Paragraph |
| --- | --- | --- | --- | --- |
| 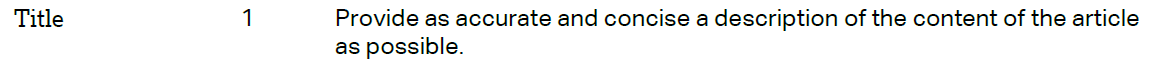 | | | Protective immunity induced by VLP vaccination in mice |  |
| 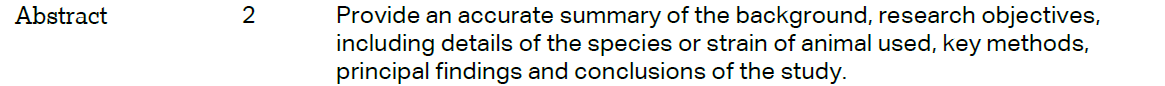 | | | Background and Purpose: Toxoplasmosis is a parasitic infection with worldwide distribution. However, there is no licensed vaccine against *T. gondii*. Our aim was to evaluate the efficacy of VLP vaccine we generated in mice.  Experimental Approach: Female BALB/c mice were immunized with VLP vaccine (IN, IM) 2 times with a 4-week interval. Four weeks after the last immunization, mice were challenged by oral administration with 1 × 10^5^ tachyzoites of the RH strain. Vaccinated mice showed a significant increase of both IgG antibodies in sera and IgA antibodies in feces compared to those before challenge, and a rapid expansion of both germinal center B cell (B220+, GL7+) and T cell (CD4+, CD8+) populations. Notably, intranasally immunized mice showed higher neutralizing antibodies and displayed no proinflammatory cytokine IFN-γ in the spleen.  Key Results: Mice were completely protected from a lethal challenge infection with the highly virulent *T. gondii* (RH) showing no body weight loss (100% survival).  Conclusion and Implication: Our study shows the effective protection against *T. gondii* infection provided by VLPs containing microneme protein 8 of *T. gondii*, thus indicating a potential *T. gondii* vaccine candidate. |  |
| INTRODUCTION | | |  |  |
| 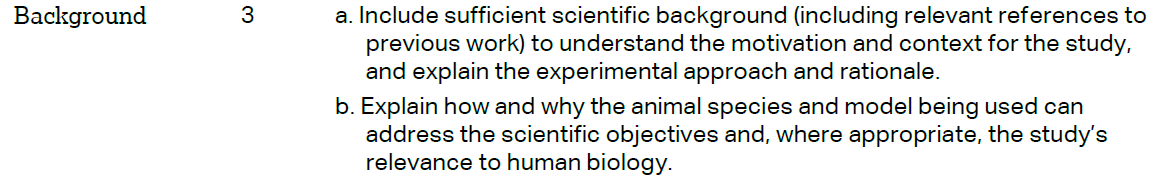 | | | 1. We have previously demonstrated the protective efficacy of virus-like particles (VLPs) containing inner membrane complex (IMC) by measuring 100% survival rate of vaccinated mice upon challenge infection [1]. However, it should be noted that ME49, a moderately virulent strain of *T. gondii*, was employed for challenge. Thus, it is critical to evaluate the effectiveness of different types of VLP vaccines against the highly virulent RH strain of *T. gondii*. Moreover, MIC8 of *T. gondii* is known to play a critical role in host cell invasion by the parasite [2].   b. We selected Balb/c mice as *T. gondii* (RH strain) infection model since it has been used successfully for decades [3,4]. |  |
| 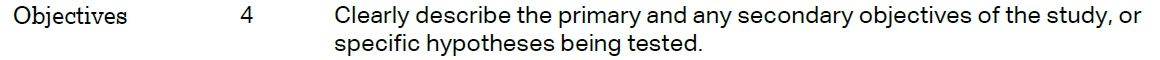 | | | The objectives of this study were to determine whether VLP vaccination induces protection against *T. gondii* infection. Therefore, it is hypothesized that *T. gondii* VLPs targeting MIC8 would elicit *T. gondii*-specific humoral and cellular immune responses, resulting in the induction of protective immunity against toxoplasmosis. |  |
| METHODS | | |  |  |
| 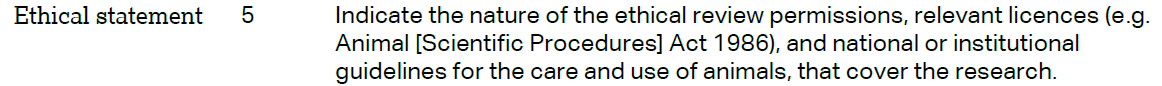 | | | All animal experiments and husbandry involved in these studies were conducted under the guidelines of the Kyung Hee University IACUC. All animal procedures performed in this study (permit number: KHUASP (SE) – 16 - 012) were reviewed, approved, and supervised by an animal research ethics committee in Kyung University. |  |
| 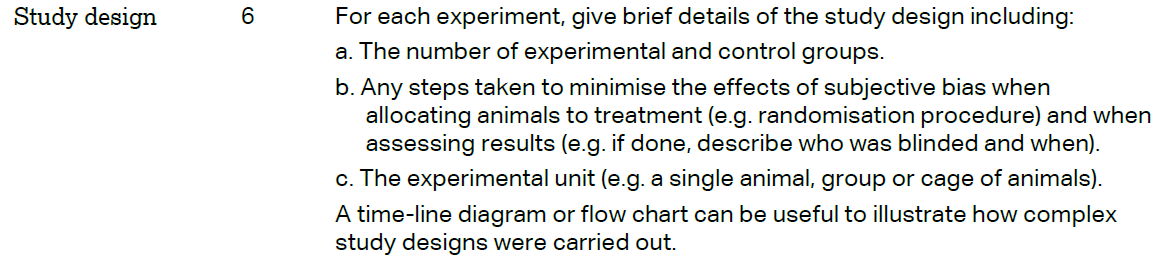 | | | 1. Four mice groups (female, BALB/c mice; 20 mice in each group) were used in this work: intranasal immunization (IN), intramuscular immunization (IM) and non-immunized controls.   b. Immunized mice were challenge infected with 1 × 10^5^ tachyzoites of the RH strain at 4 weeks after boost.  c. In the study, 10 mice were sacrificed on days 5 or 16 post-challenge, and blood and spleen samples were collected. The remaining mice (10 mice in each group) were observed daily to monitor changes in body weight and survival rates for 16 days post-challenge.  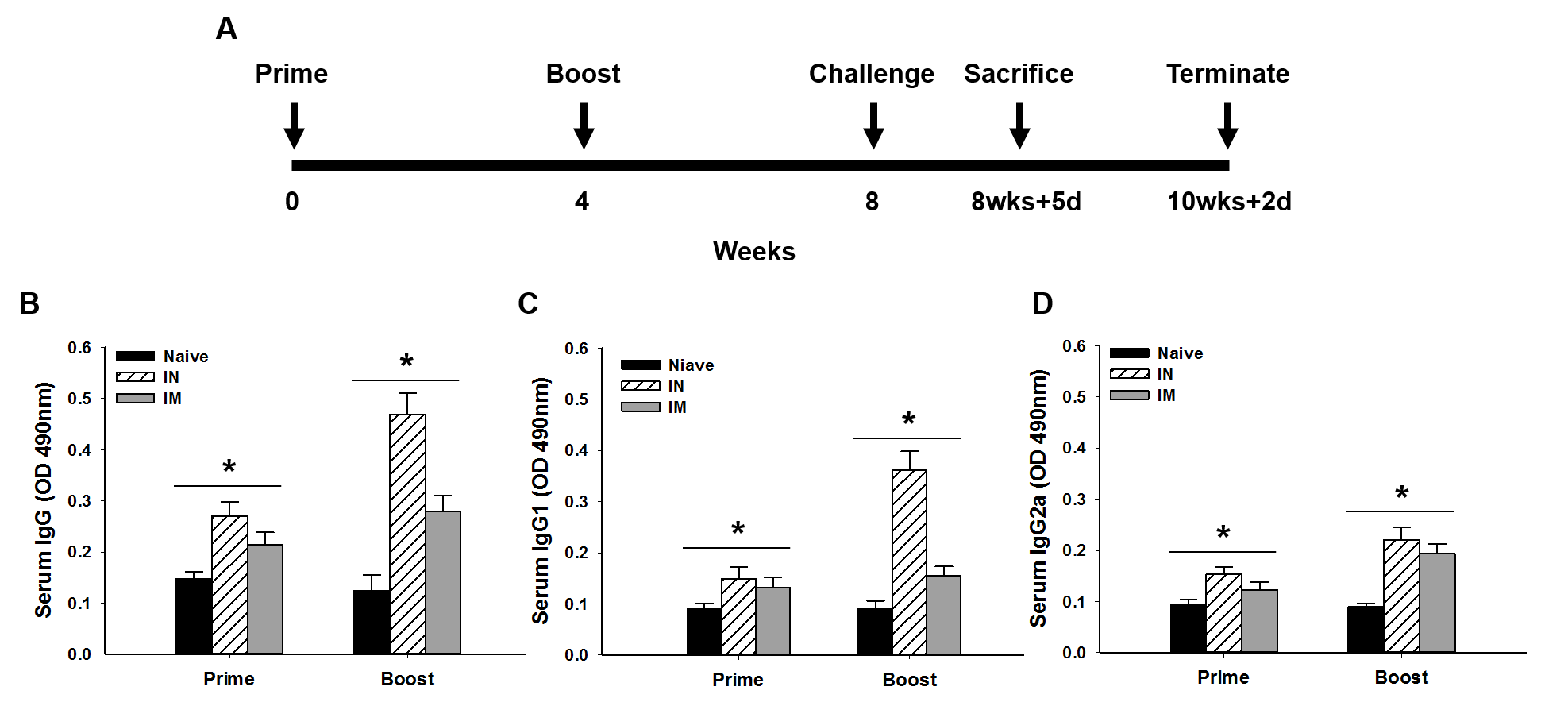  Figure 1. Experimental timelines. |  |
| 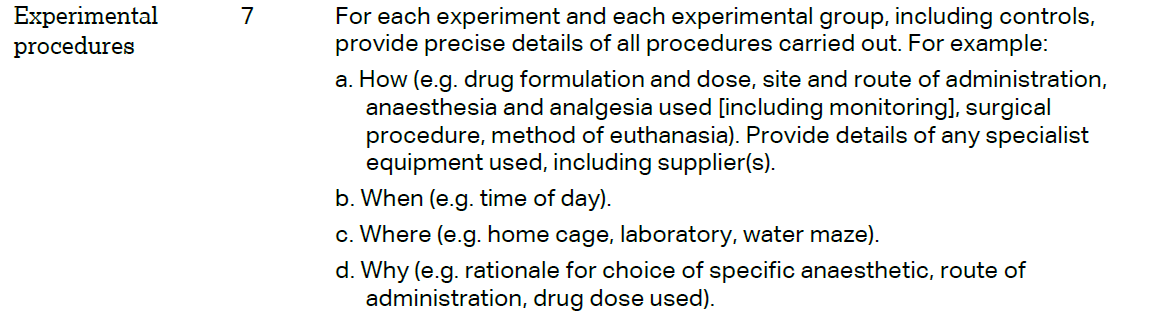 | | | Mice were immunized intranasally (IN) or intramuscularly (IM) with 75 µg of total MIC8 VLP protein per mouse at weeks 0 and 4 [1,5]. For intranasal immunization, mice were inoculated with VLPs (75 µg in 50 µL PBS) under inhalation anaesthesia. For intramuscular immunization, mice were inoculated with VLPs (75 µg in 100 µL PBS). For challenge experiment, mice were challenged by oral administration with oral gavage (22 gavage). Mice were anesthetized (isoflurane) and sacrificed on days 5 or 16 post-challenge.  All animal experiments were conducted at 10 am in the morning in the biosafety hood.  IN and IM injections are the most popular routes in evaluating vaccine efficacy [6-8]. In these vaccine studies, isoflurane (1 mL of isoflurane in 200 mL container for inhalation) and vaccine dose of 50 – 100 µg were used. The challenge dose of 1 × 10^5^ *T. condii* tachyzoites was found to be the most reasonable after we conducted mouse survival tests using different challenge doses. |  |
| 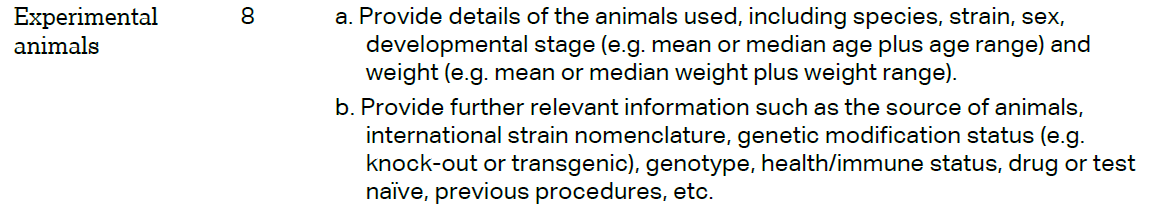 | | | Female BALB/c mice (17.4 ± 0.2 g), aged 6 - 8 weeks, were included (n = 80).  Eighty female mice were obtained from NARA Biotech (Seoul, Korea) and acclimatized for at least 72 h. [1,9]. Vendor health reports indicated that the mice were free of known viral, bacterial and parasitic pathogens. |  |

| 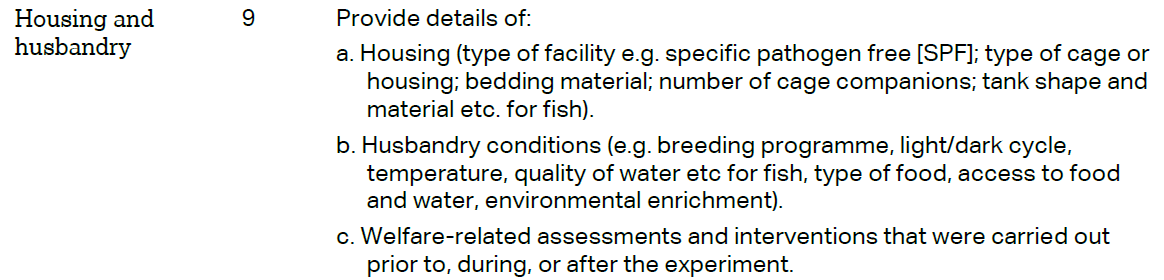 | Animals were housed with an inverse 12-hours day-night cycle with lights on at 8:30 pm in a temperature (22 ± 1 ºC) and humidity (55 ± 5%) controlled room.  All mice were allowed free access to water and a maintenance diet (Purina Diet) containing protein, fat, vitamins and minerals [10].  (<http://www.labdiet.com/cs/groups/lolweb/@labdiet/documents/web_content/mdrf/mdi4/~edisp/ducm04_028021.pdf>). This diet has been the standard of biomedical research for over 70 years.  All cages contained wood shavings and bedding for environmental enrichment. | |
| --- | --- | --- |
| 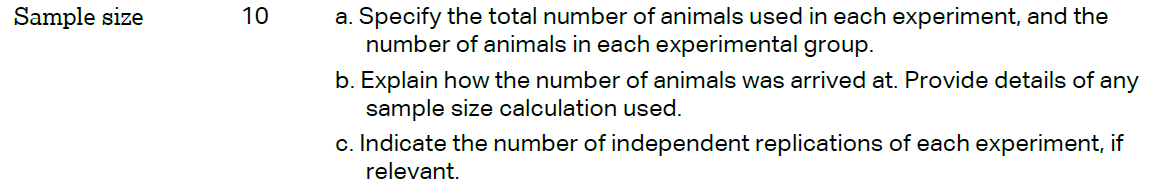 | Eighty mice were divided into 4 groups of 20 each. Naïve served as naïve control, Naïve+Cha served as naïve infection control, IM+Cha served as IM vaccinated mice challenge infected, IN+Cha served as IN vaccinated mice challenge infected [1,5].  Sample size calculations were performed using the resource equation method [11]. In this method, a value E is calculated based on the decided sample size. The value of E should lie within 10 to 20 for optimum sample size. In our study, E is 17 (20 - 3 = 17) which can be considered as appropriate sample size. This has been confirmed by power analysis method.  The experiment was repeated, and data were pooled. | |
| 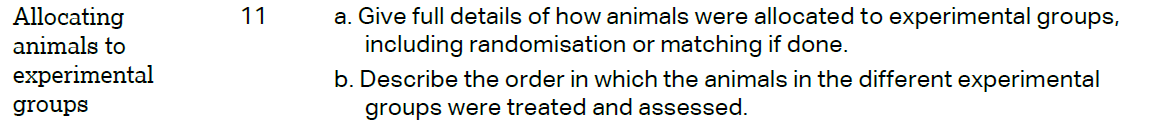 | Mice were allocated to groups randomly.  The animals in each group were marked with sequences of A-B-C during testing. | |
| 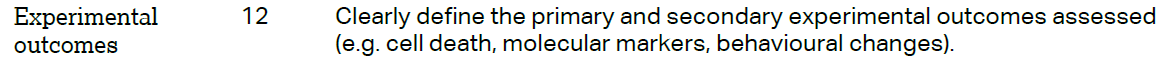 | The experimental outcomes were obtained upon prime, boost, and 5 and 16 days after challenge infection. In addition, mice body weight changes and survival were measured upon challenge infection. | |
| 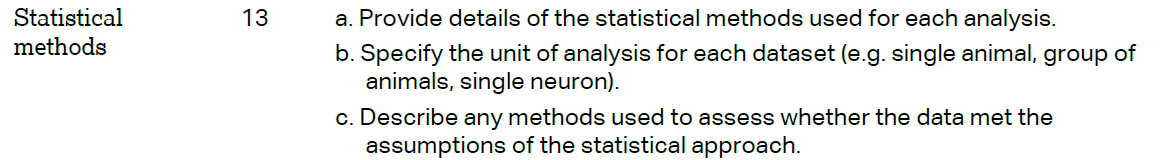 | ANOVA with multiple comparison test of PC-SAS 9.3 (SAS Institute; Cary, NC, USA) was used to analyse IgG and subclass antibody responses in serum and feces, *T. gondii* recovered from abdominal cavity, CD4, CD8 and GL7 cell populations and inflammatory cytokines.  For each test, the experimental unit was an individual animal. | |
| RESULTS |  | |
| 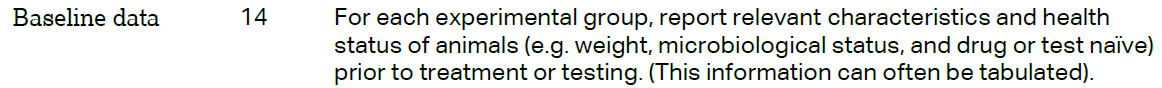 | The animals health status was monitored throughout the experiments under the guidelines of the Kyung Hee University IACUC. The mice were free of all viral, bacterial, and parasitic pathogens as listed in the vendor report. | |
| 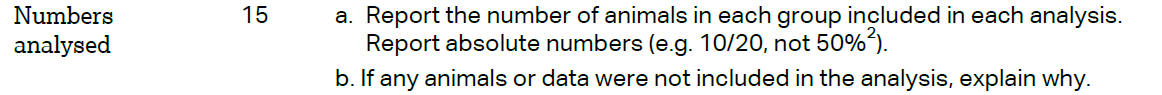 | Twenty-four samples were from D5 post-challenge (24/80 = 30%), D16 post-challenge (24/80 = 30%), and 32 mice (32/80 = 40%) were used to monitor body weight and survival daily. | |
| 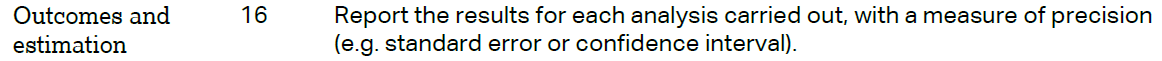 | In accordance with the ARRIVE guidelines, we have performed measures of precision, confidence, and n to provide an indication of significance in Figs 3, 4, 5, 6, 7 and 8 as indicated in the paper. | |
| 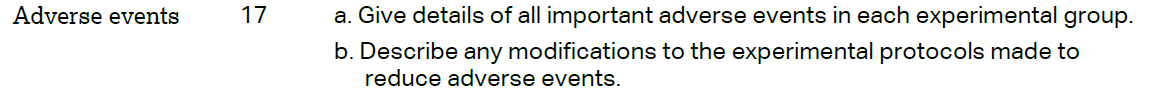 | No adverse events were found. No modification to the experimental protocol was made. | |
| DISCUSSION |  | |
| 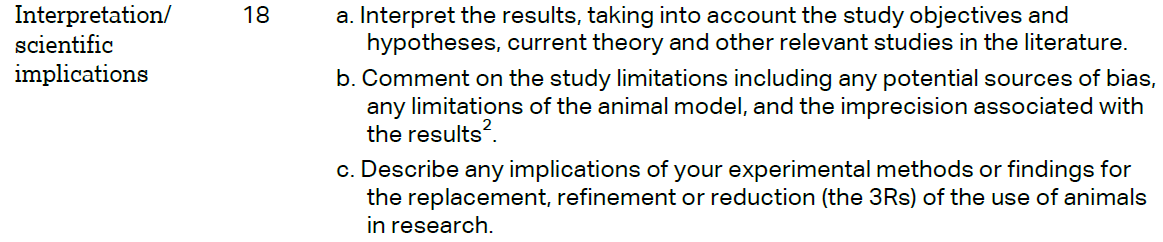 | The aim of our study was to verify whether VLP vaccination could induce protective immunity against *T. gondii* infection in mice. Mice immunized with VLPs showed significantly higher levels of antibody responses before and after challenge infections, and germinal center B cell and T cell responses. Mice showed no body weight loss (100% survival). These results indicate that VLP vaccination induces complete protection against *T. gondii* infection, thus it could be a potential *T. gondii* vaccine candidate. | |
| 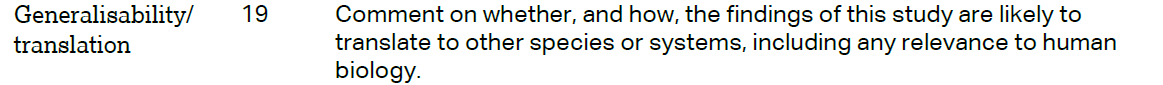 | These findings are likely to help guide the development of vaccine against *T. gondii* infection in clinical trials in the future. | |
| 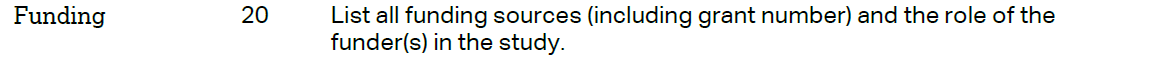 | | This work was supported by a grant from the National Research Foundation of Korea (NRF) (NRF-2014R1A2A2A01004899), a grant from the Agri-Bio Industry Technology Development Program (315030-03-1-HD020), IPET, MAFRA, KHIDI, and a grant from the Ministry of Health and Welfare, Republic of Korea (HI15C2928). |


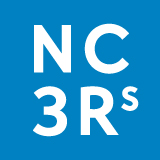


References

1. Lee DH, Lee SH, Kim AR, Quan FS. Virus-Like Nanoparticle Vaccine Confers Protection against Toxoplasma gondii. PLoS One. 2016;11: e0161231.

2. Liu M, Yuan Z, Peng G, Zhou D, He X, Yan C, et al. Toxoplasma gondii microneme protein 8 (MIC8) is a potential vaccine candidate against toxoplasmosis. Parasitol Res. 2010;106: 1079-1084.

3. Jongert E, Roberts CW, Gargano N, Förster-Waldl E, Petersen E. Vaccines against Toxoplasma gondii: challenges and opportunities. Mem Inst Oswaldo Cruz. 2009;104: 252-266.

4. Fux B, Ferreira A, Cassali G, Tafuri W, Vitor RW. Experimental toxoplasmosis in BALB/c mice. Prevention of vertical disease transmission by treatment and reproductive failure in chronic infection. Memorias do Instituto Oswaido Cruz. 2000;95: 121-126.

5. Lee D, Kim A, Lee S, Quan F. Cross-protection induced by Toxoplasma gondii virus-like particle vaccine upon intraperitoneal route challenge. Acta Trop. 2016;164: 77-83.

6. Quan FS, Steinhauer D, Huang C, Ross TM, Compans RW, Kang S. A bivalent influenza VLP vaccine confers complete inhibition of virus replication in lungs. Vaccine. 2008;26: 3352-3361.

7. Quan FS, Yoo DG, Song JM, Clements JD, Compans RW, Kang SM. Kinetics of immune responses to influenza virus-like particles and dose-dependence of protection with a single vaccination. J Virol. 2009;83: 4489-4497.

8. Quan F, Vunnava A, Compans RW, Kang S. Virus-like particle vaccine protects against 2009 H1N1 pandemic influenza virus in mice. PLoS One. 2010;5: e9161.

9. Chu KB, Kim SS, Lee SH, Lee DH, Kim AR, Quan FS. Immune Correlates of Resistance to Trichinella spiralis Reinfection in Mice. Korean J Parasitol. 2016;54: 637-643.

10. LabDiet.; 2013 . Database: figshare [Internet]. Available from:
:<http://www.labdiet.com/cs/groups/lolweb/@labdiet/documents/web_content/mdrf/mdi4/~edisp/ducm04_028021.pdf>.

11. Charan J, Biswas T. How to calculate sample size for different study designs in medical research? Indian J Psychol Med. 2013;35: 121-126.
